# Characteristics of carbohydrate counting practice associated with adequacy of glycated hemoglobin in adults with type 1 diabetes mellitus in Brazil

**DOI:** 10.3389/fendo.2023.1215792

**Published:** 2023-09-11

**Authors:** Gabriela Correia Uliana, Lediane Nunes Camara, Carla Cristina Paiva Paracampo, Juliana Carvalho da Costa, Daniela Lopes Gomes

**Affiliations:** ^1^ Nucleus of Behavior Theory Research, Federal University of Pará, Belém, Brazil; ^2^ Faculty of Nutrition, Federal University of Pará, Belém, Brazil

**Keywords:** diabetes mellitus, carbohydrates, glycated hemoglobin, treatment adherence, adults

## Abstract

**Background:**

The Carbohydrate Counting (CC) is directly associated with achieving glycemic control by people with Type 1 Diabetes Mellitus (T1DM). Therefore, this study aims to analyze characteristics of the CC practice associated with the adequacy of glycated hemoglobin (HbA1c) in adults with T1DM in Brazil.

**Methods:**

The study was cross-sectional, carried out using an online form with questions about knowledge of CC, clinical, anthropometric, sociodemographic data, follow-up with health professionals and understanding of the concepts of CC. Pearson’s chi-square test and binomial logistic regression analysis (p<0.05) were applied.

**Results:**

173 adults participated, of which 57.2% had increased HbA1c (≥7%). Having the diabetes duration <10 years (p=0.006), performing the CC at lunch (p=0.040) and dinner (p=0.018), using specific applications to perform the CC (p=0.001), having learned to perform CC with a nutritionist (p=0.037) and knowing how to correctly define the concepts of food bolus (p=0.001), correction bolus (p<0.001) and insulin/carbohydrate ratio (p<0.001) was associated with having adequate HbA1c (<7%). Participants who were undergoing CC practice were 3.273 times more likely to have adequate HbA1c and participants with diabetes duration <10 years were 2.686 times more likely to have adequate HbA1c.

**Conclusion:**

It was concluded that variables transversal to CC favor adequate HbA1c values in adults with T1DM and that practicing CC and having a diabetes duration of less than 10 years are predictive factors of having adequate HbA1c.

## 1 Introduction

In Brazil, the estimation for incident and prevalent cases in individuals with Type 1 Diabetes Mellitus (T1DM) aged less than 20 years is 8,900 and 92,300, respectively, thus occupying the third place in the ranking of countries with the highest rates of incident and prevalent cases in this age group (1). Although the diagnosis of T1DM is more common in childhood and adolescence, it can also occur in adulthood (2, 3). However, there is still no estimation of the number of incident cases in people with onset of T1DM in adulthood in Brazil.

T1DM has hyperglycemia as a clinical manifestation, as a result of the deficiency or absence of insulin production by the pancreas, caused by the destruction of pancreatic beta cells. As a result, the main objective of treatment is to achieve and maintain glycemic control in patients, in order to prevent possible complications from the disease and ensure a longer and healthier life expectancy (3–6).

Therefore, it is necessary to have a continuous treatment with a high response cost, based on the behavior of applying multiple doses of exogenous insulin throughout the day, regularly monitoring blood glucose, practicing physical activity and consuming a healthy diet (7). Adherence to a healthy diet is the basis for all other pillars of treatment to work properly, however, determining what to eat is the most costly task of the treatment plan for many patients with diabetes (4).

Moreover, the professional nutritionist, who has specific knowledge and skills for managing diabetes, plays a fundamental role throughout the treatment, as there is no specific eating pattern for this public, and it is essential that the patient himself participate in the construction of the food plan, so that it is prepared individually, considering the culture, financial condition, personal preferences and comorbidities of the patient (4, 5, 7, 8).

In addition to the traditional dietary prescription model, there are other strategies that help reduce glycemic variability in patients with T1DM, such as Carbohydrate Counting (CC), which has been recognized since 1993 for providing flexibility in food choices and ensuring better quality of life (9–11).

CC involves balancing the amount of carbohydrates ingested, the dose of insulin administered and the blood glucose value, and it can be performed in two ways. The first is based on grouping foods based on their nutritional composition, in portions that correspond to approximately 15 grams of carbohydrates, allowing for adjustments between foods in the same group (12–14). The second approach is more accurate, as it involves summing up the total carbohydrate grams of a meal, either by weighing, home measuring, or nutritional information on labels, allowing for the administration of bolus insulin based on the amount of carbohydrates consumed (7, 12).

Based on this, CC contributes to the management and control of carbohydrate intake, which is directly associated with achieving glycemic control, as carbohydrate is the macronutrient that most impacts the variation in blood glucose levels, as it is completely converted into glucose in the bloodstream (11, 15, 16).

Glycemic control involves measures such as fasting blood glucose, pre- and postprandial blood glucose, and glycated hemoglobin (HbA1c). Blood glucose values throughout the day help in monitoring medications, correcting hyper or hypoglycemia, and understanding the effects of food, stress, emotions and physical exercise on glycemia. HbA1c reflects the average blood glucose levels over the last three or four months, with values below 7% being recommended to prevent long-term microvascular and macrovascular complications. Therefore, self-monitoring of capillary glycemia and HbA1c are complementary to glycemic control (7, 17, 18).Studies already show the benefits of CC on HbA1c values and glycemic control. In the systematic review and meta-analysis performed by Vaz et al. (19) it was found that HbA1c, at the end of the analyzed studies, was significantly lower in groups that used CC than in control groups, which received conventional nutritional guidance and used fixed doses of insulin before meals. Likewise, Donzeau et al. (20) observed, during the one-year intervention period with children and adolescents, that the average HbA1c at three, six, nine and twelve months was lower in the group that received CC education compared to the control group, which received traditional eating education.

In the study by Kostopoulou et al. (21), also carried out with children and adolescents, it was possible to observe that there was statistical significance between having worse glycemic control, based on HbA1c values, and using fixed-dose insulin therapy, compared to applying insulin doses according to the CC. The concentrations of ferric reducing antioxidant power, used as a marker of antioxidant capacity, were also significantly higher after the use of CC, which suggests a better impact on antioxidant defense mechanisms by obtaining a better glycemic profile. In addition, it was observed that CC favors the reduction of the malondialdehyde biomarker, which is a powerful biomarker of oxidative stress and an intermediary of complications in T1DM. Altogether, these results suggest that CC is an important strategy to be implemented in the treatment of patients with T1DM, preferably in the initial period of diagnosis, with the aim of achieving early glycemic control.

Although studies have already shown the benefits of CC on HbA1c values, there is still a scarcity of studies that analyze variables cross-sectional to CC that may interfere with HbA1c values, such as the means used for adherence, follow-up with health professionals and understanding of strategy concepts. Thus, the objective of this study was to analyze characteristics of the practice of CC associated with the adequacy of HbA1c in adults with T1DM in Brazil, as well as to verify the relationship between the practice of CC, the time of diagnosis and the achievement of adequate levels of HbA1c.

## 2 Materials and methods

### 2.1 Type of Study

This is a cross-sectional, descriptive and analytical study, carried out between November 2021 and June 2022. The research was disseminated through social networks (WhatsApp^®^, Facebook^®^ and Instagram^®^) of the researcher and the Extension Project “Grupo Educativo em Diabetes [Diabetes Education Group]”, which is linked to the Faculty of Nutrition of a public university in northern Brazil.

### 2.2 Participants

A convenience sampling was carried out with 173 adults. Inclusion criteria were having a diagnosis of T1DM, age between 18 and 59 years old, of both genders, knowing how to perform the Carbohydrate Counting (CC) and agreeing to participate in the research voluntarily and anonymously, by checking the alternative “I have read the FICF and I AGREE to participate in the research”, displayed after reading the Free and Informed Consent Form (FICF). Data from people who did not fit the inclusion criteria or who did not complete the survey were excluded. If they clicked on the option “I do not accept to participate in the research”, after reading the FICF, the research was closed.

### 2.3 Instrument

An online questionnaire was used, built on the Google Forms^®^ platform, consisting of 31 questions, 26 objective and five simple subjective questions (weight, height, HbA1c value, age and time since diagnosis, in years), in the research format of opinion, according to Resolution 510 of April 7, 2016 (22). The questions were divided into five axes, namely:

(a) Knowledge of the CC practice: Containing questions regarding knowing how to do the CC; at what time do you do the CC; what means do you use to check the amount of carbohydrates in food; if you use a kitchen scale to do the CC; and why you use the kitchen scale;
(b) Clinical and anthropometric data: Containing questions regarding the time of diagnosis; weight; height; HbA1c test; and HbA1c value in the last test performed by the participants;
(c) Sociodemographic and socioeconomic data: Containing questions related to age; biological gender; country’s region; level of education and family income;
(d) Monitoring with health professionals (considering the three months prior to the survey): Questions about whether it is attended in person, virtually, by both, or if it is not attended; whether monitoring is carried out through a health plan/insurance, through the SUS, through both, or privately; and who taught you how to do the CC;
(e) Perception of difficulties related to understanding the concepts of CC as a limitation: Questions to check whether participants know the concepts of food bolus, correction bolus, sensitivity factor, and insulin/carbohydrate ratio; questions in which it is necessary to mark the alternative that correctly defines the concept of food bolus, correction bolus, sensitivity factor, and insulin/carbohydrate ratio; and finally, questions about the participant’s perception of the influence of understanding the concepts on performing the CC.

The construction of the questionnaire was based on a preliminary study carried out by Uliana et al. (23), which aimed to analyze sociodemographic and treatment factors associated with adherence to the CC strategy by adults with T1DM during social distancing in the COVID-19 pandemic in Brazil.

The questionnaire was submitted to two types of validation, content and appearance. Content validity consists of checking whether the concepts addressed within the tool are adequate, whether they are presented correctly, as well as analyzing whether the items are relevant within the product universe. While the appearance validity corresponds to subjectively evaluating an instrument or strategy, comprising the judgment stage regarding clarity and understanding (24, 25).

In the present research, the content validation was carried out first, and then the appearance validation, through the evaluation of three specialist judges. All judges were nutritionists, working in the state of Pará, Brazil, and were qualified in diabetes education by the SBD/ADJ/IDF (Brazilian Diabetes Society/Juvenile Diabetes Association/International Diabetes Federation), one had a doctorate degree and two had master’s degree.

### 2.4 Proceeding

Initially, the survey link was sent directly to people who claimed to have T1DM in their bio on social networks (Whatsapp^®^, Facebook^®^ and Instagram^®^). By clicking on the available link, the person was directed, first, to the summary explanation of the study. Then, the link was provided, specifying that no type of identification of the participants would be required. After reading the FICF, the person agreed or not to participate. If the person clicked on the option “I read the FICF and I AGREE to participate in the research”, the person was forwarded to the page where the other inclusion criteria were applied.

The participants who marked the alternatives referring to knowing how to do CC, present in the first question of axis 1 of the form, were directed to the questions referring to each axis, following the order: 1) Knowledge of the CC practice; 2) Clinical and anthropometric data; 3) Sociodemographic and socioeconomic data; 4) Monitoring with health professionals; and 5) Perception of difficulties related to understanding the concepts of CC as a limitation. The participant was only forwarded to the next axis if he had answered all the questions in the previous axis. At the end, they were directed to the closing page of the research, which contained the link to the Carbohydrate Counting Manual for People with Diabetes, prepared by the Brazilian Diabetes Society, SBD (11), which contains instructions for performing the CC, in addition to a table with the amount of carbohydrates in different kinds of food.

### 2.5 Data analysis

For statistical analysis, the Statistical Package for Social Science software, version 24.0, was used. Descriptive results were expressed in absolute frequency and proportion. In the analytical stage, Pearson’s Chi-Square test was applied, considering the sample n and sample distribution respectively. The level of statistical significance considered was p<0.05.

Before the logistic regression analysis, the absence of co-linearity between the study variables was observed through linear regression, observing tolerance and VIF values, all of which were greater than 0.1 and less than 10, respectively. Finally, the binomial logistic regression analysis was performed, consisting of having adequate or increased HbA1c according to the reference values (dependent variable) and practicing or not practicing CC (does or does not practice it) and time since diagnosis (<10 years and >10 years) (independent variables). The reference values for HbA1c were <7% for adequate HbA1c and >7% for increased HbA1c, following the guidelines of the American Diabetes Association and Brazilian Diabetes Society (17, 18). For the independent variable to practice or not to practice CC (does or does not practice it), those who checked the option “I do the CC” in the research questionnaire were considered CC practitioners. Those who checked the options “I already did, but currently I’m not doing it” and “I know how to do it, but I’ve never done the CC” were considered non-practitioners of CC. The final model was able to predict 64.2% of the adequacy of HbA1c in the studied sample.

### 2.6 Ethical aspects

The research complied with the legal requirements of Resolutions 466 of December 12, 2012, and 510 of April 7, 2016, published by the National Health Council, which considers the Declaration of Helsinki for studies involving human beings (22, 26).

The conduction of the study was approved by the Research Ethics Committee from the Nucleus of Tropical Medicine of the Federal University of Pará - opinion n. 5.077.488 and CAAE 51974621.7.0000.5172.

## 3 Results

The survey was answered by 260 people, of which 173 met the inclusion criteria of the study, the majority were female (84.4%), aged between 25 and 44 years (67.1%), residing in the Southeast region of Brazil (30.1%), completed higher education (52.6%), had a family income of more than three minimum wages (55.5%), had an increased HbA1c value (57.2%), with an average of 7.3 ± 1.3% and mean duration of diabetes of 15.50 ± 9.10 years.

When checking the average HbA1c according to nutritional status, according to BMI, it was observed that the average HbA1c was 9.07 ± 1.87% for participants classified as malnourished, while for participants classified as eutrophic, the average was 7.10 ± 1.16% and for overweight participants it was 7.54 ± 1.34%. When separating the groups referring to the time of diagnosis < 10 years and > 10 years, it was observed that the mean duration of diabetes in the group with < 10 years of diagnosis was 5.04 ± 2.95 years, in the group with > 10 years was 19.64 ± 7.19 years.

When analyzing the associations between socioeconomic and demographic data and adequacy of HbA1c, it was observed that there was no association between having adequate or increased HbA1c and gender, age, country’s region of residence, education and family income of the participants (**Table 1**).

**Table 1:** Association between socioeconomic, demographic, and clinical data and adequacy of HbA1c in adults with T1DM in Brazil, 2022.

	HbA1c		p- value*
	Adequate	Increased	
	n (%)	n (%)	
Sex			
Male	10 (5.8)	17 (9.8)	0.512
Female	64 (37.0)	82 (47.4)
Age (years old)			
18 – 24	21 (12.1)	30 (17.3)	0.844
25 – 44	51 (29.5)	65 (37.6)
45 – 59	2 (1.2)	4 (2.3)
Country’s region			
North	2 (1.2)	8 (4.6)	0.441
Northeast	16 (9.2)	20 (11.6)
Midwest	20 (11.6)	19 (11.0)
Southeast	20 (11.6)	32 (18.5)
South	16 (9.2)	20 (11.6)
Education			
No higher education	32 (18.5)	50 (28.9)	0.344
Higher education	42 (24.3)	49 (28.3)
Family Income**			
Up to 3 MW	29 (16.8)	48 (27.7)	0.224
More than 3 MW	45 (26.0)	51 (29.5)
HbA1c measurement time			
Until 3 months ago	54 (31.2)	62 (35.8)	0.152
More than 3 months ago	20 (11.6)	37 (21.4)
BMI Classification Categories			
Eutrophic	50 (28.9)	54 (31.2)	0.033 ^†^
Malnutrition	0 (0.0) (-)	7 (4.0) (+)
Overweight	24 (13.9)	38 (22.0)
Classification of diagnostic time			
Less than 10 years	29 (16.8) (+)	20 (11.6) (-)	0.006 ^†^
More than or equal to 10 years	45 (26.0) (-)	79 (45.7) (+)

*Chi-square. ^†^ Statistical Significance; Residue Analysis: (+) Significant Association (−) Negative Significant Association; HbA1c, Glycated hemoglobin; BMI, Body Mass Index; MW, Minimum Wage; **Minimum Wage = R$1,100.00.

Regarding the adequacy of HbA1c and clinical data, having malnutrition (p = 0.033), according to BMI, and more than 10 years of diagnosis (p = 0.006) was associated with having increased HbA1c, while having a diagnosis time of less than 10 years was associated with having adequate HbA1c (p = 0.006) (**Table 1**).

Having adequate HbA1c was associated with practicing CC (p = 0.007), practicing CC at lunch (p = 0.040) and dinner (p = 0.018), using CC-specific (p = 0.001) and non-CC-specific (p = 0.001) apps to research the amount of carbohydrate in food, use the food scale to practice the CC only when eating something different than usual (p = 0.009) and use the scale to practice the CC (p = 0.001) and cooking recipes (p = 0.001). However, having already practiced CC but not currently practicing it (p = 0.007), not having it (p = 0.009) and not using a food scale to practice CC (p = 0.006) was associated with having an increased HbA1c (**Table 2**).

**Table 2:** Association between adherence to CC and adequacy of HbA1c in adults with T1DM in Brazil, 2022.

	HbA1c		p- value*
	Adequate	Increased	
	n (%)	n (%)	
Adherence to the CC			
I do the CC	63 (36.4) (+)	63 (36.4) (-)	0.007 ^†^
I already did, but currently I’m not doing it	10 (5.8) (-)	31 (17.9) (+)
I know how to do it, but I’ve never done the CC	1 (0.6)	5 (2.9)
Practicing CC in meals			
Breakfast	65 (37.6)	80 (46.2)	0.214
Morning snack	48 (27.7)	52 (30.1)	0.104
Lunch	68 (39.3) (+)	80 (46.2) (-)	0.040 ^†^
Afternoon snack	56 (32.4)	66 (38.2)	0.199
Dinner	67 (38.7) (+)	76 (43.9) (-)	0.018 ^†^
Supper	39 (22.5)	51 (29.5)	0.877
Other meals	28 (16.2)	33 (19.1)	0.540
In no meal	7 (4.0)	16 (9.2)	0.199
Means used to research the amount of carbohydrate			
Printed table	17 (9.8)	36 (20.8)	0.059
Online table	30 (17.3)	41 (23.7)	0.908
CC-specific apps	48 (27.7) (+)	39 (22.5) (-)	0.001 ^†^
Non-CC-specific apps	35 (20.2) (+)	22 (12.7) (-)	0.001 ^†^
Google and other sites	23 (13.3)	27 (15.6)	0.585
Guess the amount of carbohydrate	36 (20.8)	48 (27.7)	0.983
Does not use any means	1 (0.6)	6 (3.5)	0.120
Using the food scale to do the CC			
Yes, I use it with every meal	8 (4.6)	9 (5.2)	0.009 ^†^
Yes, I use it when I eat something different than usual	17 (9.8) (+)	9 (5.2) (-)
Yes, I use it in some meals	32 (18.5)	34 (19.7)
No, I don’t have a food scale	11 (6.4) (-)	34 (19.7) (+)
No, I don’t think it’s necessary	6 (3.5)	13 (7.5)
Why use the food scale			
To practice the CC	54 (31.2) (+)	47 (27.2) (-)	0.001 ^†^
To cooking recipes	29 (16.8) (+)	17 (9.8) (-)	0.001 ^†^
To count calories	17 (9.8)	12 (6.9)	0.059
Does not use the food scale	18 (10.4) (-)	44 (25.4) (+)	0.006 ^†^

*Chi-square. ^†^ Statistical Significance; Residue Analysis: (+) Significant Association (−) Negative Significant Association; HbA1c, Glycated hemoglobin; CC, Carbohydrate counting.

It was observed that having learned to perform CC from a nutritionist (p = 0.037) or another health professional (p = 0.004) was associated with having adequate HbA1c. There was no association between the adequacy of HbA1c and how consultations were carried out in the last three months and the form of access to monitoring with health professionals (**Table 3**).

**Table 3:** Association between follow-up with health professionals and adequacy of HbA1c in adults with T1DM in Brazil, 2022.

	HbA1c		p- value*
	Adequate	Increased	
	n (%)	n (%)	
In the last three months, I had consultations			
Face-to-face	45 (26.0)	55 (31.8)	0.312
By Internet	6 (3.5)	3 (1.7)
Face-to-face and via the internet	10 (5.8)	19 (11.0)
I had no consultation	13 (7.5)	22 (12.7)
Follow up with health professionals			
Health plan/insurance	26 (15.0)	27 (15.6)	0.060
Unified Health System (SUS)	9 (5.2)	29 (16.8)
Both (Health Plan/Insurance and SUS)	16 (9.2)	16 (9.2)
Private service	23 (13.3)	27 (15.6)
Health professional who taught how to do CC			
Endocrinologist	42 (24.3)	66 (38.2)	0.183
General Practitioner	2 (1.2)	3 (1.7)	0.899
Nutritionist	44 (25.4) (+)	43 (24.9) (-)	0.037 ^†^
Nurse	2 (1.2)	5 (2.9)	0.438
Other	29 (16.8) (+)	19 (11.0) (-)	0.004 ^†^

*Chi-square. ^†^ Statistical Significance; Residue Analysis: (+) Significant Association (−) Negative Significant Association; HbA1c, Glycated hemoglobin; CC, Carbohydrate counting.

Regarding the CC concepts, knowing what food bolus is (p = 0.001), correction bolus (p = 0.020) and sensitivity factor (p = 0.045), and knowing how to correctly define food bolus concepts (p = 0.001), bolus correction (p < 0.001) and insulin/carbohydrate ratio (p < 0.001), was associated with having adequate HbA1c. Furthermore, considering that not knowing what the insulin/carbohydrate ratio is does not influence the lack of adherence to CC (p = 0.023) was also associated with having adequate HbA1c (**Table 4**).

**Table 4:** Association between understanding the concepts of CC and adequacy of HbA1c in adults with T1DM in Brazil, 2022.

	HbA1c		p- value*
	Adequate	Increased	
	n (%)	n (%)	
Knowing what a “food bolus” is			
I’ve heard of it, but I don’t know what it is	4 (2.3) (-)	15 (8.7) (+)	0.001 ^†^
Yes, I know what it is	68 (39.3) (+)	68 (39.3) (-)
I do not know what is	2 (1.2) (-)	16 (9.2) (+)
Definition of “food bolus”			
Correctly defined	59 (34.1) (+)	56 (32.4) (-)	0.001 ^†^
Wrongly defined	15 (8.7) (-)	43 (24.9) (+)
Knowing what a “correction bolus” is			
I’ve heard of it, but I don’t know what it is	3 (1.7)	11 (6.4)	0.020 ^†^
Yes, I know what it is	66 (38.2) (+)	71 (41.0) (-)
I do not know what is	5 (2.9) (-)	17 (9.8) (+)
Definition of “correction bolus”			
Correctly defined	60 (34.7) (+)	55 (31.8) (-)	< 0.001 ^†^
Wrongly defined	14 (8.1) (-)	44 (25.4) (+)
Knowing what a “sensitivity factor” is			
I’ve heard of it, but I don’t know what it is	3 (1.7)	11 (6.4)	0.045 ^†^
Yes, I know what it is	68 (39.3) (+)	77 (44.5) (-)
I do not know what is	3 (1.7)	11 (6.4)
Definition of “sensitivity factor”			
Correctly defined	53 (30.6)	57 (32.9)	0.057
Wrongly defined	21 (12.1)	42 (24.3)
Knowing what a “insulin/carbohydrate ratio” is			
I’ve heard of it, but I don’t know what it is	6 (3.5)	9 (5.2)	0.057
Yes, I know what it is	64 (37.0)	73 (42.2)
I do not know what is	4 (2.3)	17 (9.8)
Definition of “insulin/carbohydrate ratio”			
Correctly defined	46 (26.6) (+)	32 (18.5) (-)	< 0.001 ^†^
Wrongly defined	28 (16.2) (-)	67 (38.7) (+)
Considers that not knowing what is “food bolus” influences the lack of adherence to CC			
Yes, it influences a lot	46 (26.6)	66 (38.2)	0.771
Yes, it influences a little	16 (9.2)	19 (11.0)
Does not influence	8 (4.6)	7 (4.0)
I don’t know	4 (2.3)	7 (4.0)
Considers that not knowing what “bolus correction” is influences the lack of adherence to CC			
Yes, it influences a lot	41 (23.7)	60 (34.7)	0.855
Yes, it influences a little	21 (12.1)	24 (13.9)
Does not influence	6 (3.5)	6 (3.5)
I don’t know	6 (3.5)	9 (5.2)
Considers that not knowing what a “sensitivity factor” is influences the lack of adherence to CC			
Yes, it influences a lot	42 (24.3)	60 (34.7)	0.338
Yes, it influences a little	21 (12.1)	25 (14.5)
Does not influence	7 (4.0)	4 (2.3)
I don’t know	4 (2.3)	10 (5.8)
Considers that not knowing what the “insulin/carbohydrate ratio” is influences the lack of adherence to CC			
Yes, it influences a lot	46 (26.6)	67 (38.7)	0.023 ^†^
Yes, it influences a little	17 (9.8)	18 (10.4)
Does not influence	8 (4.6) (+)	2 (1.2) (-)
I don’t know	3 (1.7)	12 (6.9)

*Chi-square. ^†^ Statistical Significance; Residue Analysis: (+) Significant Association (−) Negative Significant Association; HbA1c, Glycated hemoglobin; CC, Carbohydrate counting.

On the other hand, having heard about it, but not being able to say what it is and not knowing what a food bolus is (p = 0.001), not knowing what a correction bolus is (p = 0.020), not knowing how to correctly define the concept of a food bolus (p = 0.001), bolus correction (p < 0.001) and insulin/carbohydrate ratio (p < 0.001) were associated with increased HbA1c (**Table 4**).

Practicing CC and having been diagnosed for less than 10 years was a significant predictor of having adequate HbA1c. Participants who were practicing CC were 3,273 times more likely to have adequate HbA1c and participants with a diagnosis time of less than 10 years were 2,686 times more likely to have adequate HbA1c (**Table 5**).

**Table 5:** Binomial logistic regression between adherence to CC, time of diagnosis and adequacy of HbA1c in adults with T1DM in Brazil.

	B	S.E.	Wald	df	Sig.	OR (odds ratio)*	95% C.I. for EXP(B)	
							Lower	Upper
Adherence to CC	1.186	0.388	9.344	1	0.002	3.273	1.530	6.999
Time of diagnosis	0.988	0.359	7.556	1	0.006	2.686	1.328	5.433
Constant	-1.186	0.496	5.722	1	0.017	0.306		

*OR- odds ratio (OR = eβ); Binomial logistic regression. Dependent variable: Adequacy of Glycated Hemoglobin; Independent variables: Adherence to Carbohydrate Counting (yes or no) and time of diagnosis (<10 years and >10 years).

It was observed with the linear regression that practicing CC is a predictive factor for having adequate HbA1c, regardless of the time of diagnosis (Beta=0.298; p=0.000; CI 0.454 – 1.308) (**Table 6**).

**Table 6:** Linear regression between adherence to CC, time of diagnosis and adequacy of HbA1c in adults with T1DM in Brazil.

Model		Unstandardized Coefficients		Standardized Coefficients	t	Sig.	CI 95%	
		B	Std. Error	Beta			Lower Bound	Upper Bound
(Constant)		6.219	0.291		21.384	0.000	5.645	6.793
To do or not to CC		0.881	0.216	0.298	4.081	0.000	0.455	1.307
(Constant)		6.150	0.335		18.361	0.000	5.489	6.811
To do or not to CC		0.881	0.216	0.298	4.073	0.000	0.454	1.308
Time of diagnosis		0.004	0.011	0.031	0.417	0.677	-0.017	0.025

Linear regression. Dependent variable: Adequacy of Glycated Hemoglobin; Independent variables: Adherence to Carbohydrate Counting (to do or not to CC) and time of diagnosis (<10 years and >10 years).

## 4 Discussion

In the present study, it was possible to observe a high percentage of participating women (84.4%). Data from the Ministry of Health show that the frequency of medical diagnosis of diabetes is higher in women (9.6%) compared to men (8.6%) in Brazil, with no distinction between types of diabetes (27). In the epidemiological study by Maahs et al. (28), T1DM affects men and women equally. However, in 2020, Uliana et al. (23) conducted an online survey with adults with T1DM and observed a high prevalence of female respondents (86.0%). The same occurred in the online survey conducted by Fortin et al. (29) with adults with T1DM, in which the majority of participants were women (64%). Kemp (30), prepared a report to understand how people used connected devices and services in Brazil in 2022, showing that in the first half of the year more than 50% of the advertisement audience on the social networks Facebook^®^ and Instagram^®^ (53.6% and 58.7%, respectively) was female. Thus, there is the hypothesis that the participation of women is greater in social networks, which may explain the predominance of this public in online surveys.

Regarding the mean value of HbA1c, it was observed that more than half of the participants had increased HbA1c, with a mean value of 7.3 ± 1.3%. Donzeau et al. (20) found a reduction in the mean HbA1c of children and adolescents who practiced CC compared to children who practiced traditional eating education during the one-year intervention period of the study (7.63 ± 0.43 vs. 7.85± 0.47%). In the same way that Kostopoulou et al. (21) identified a reduction in HbA1c values in children and adolescents four months after starting CC practice (HbA1c = 7.40%) compared to the previous four months using standard doses of insulin (HbA1c = 7.90%). Added to this, in the systematic review and meta-analysis by Bell et al. (31) it was observed that five of the seven studies analyzed favored CC. The five adult studies with a parallel design, which compared CC with alternative advice or usual care, showed a 0.64% reduction in HbA1c in the CC groups when compared to HbA1c concentration at baseline and at the end of the intervention.

Although no study with CC has found HbA1c values within the values recommended by the SBD (Brazilian Diabetes Society) and ADA (American Diabetes Association), which are HbA1c < 7.0% for people with T1DM, in order to prevent microvascular and macrovascular complications, it is possible to observe that CC helps in the reduction of these values compared to the non-application of the strategy (17, 18). From this, the importance of CC to help glycemic control is reiterated and the need to implement the strategy is reinforced through the consumption of a healthy diet and with the monitoring of trained nutritionists (7, 11, 13, 14).

Furthermore, an association was found between having an increased HbA1c and having malnutrition according to BMI, which may be related to an imbalance in food consumption and, consequently, calories, due to fear of increased blood glucose, resulting in uncontrolled glycemic control, ketosis and other complications important for nutritional status (32–34). Another factor that can interfere with the nutritional status of patients with DM1 is diabulimia, a term that characterizes the omission of insulin doses in order to lose weight (35, 36). In the study by Coleman & Caswell (37) a high percentage (78%) of participants with DM1 who declared weight loss as the main reason for insulin restriction was observed. Other studies have also shown the behavior of restricting insulin by patients with DM1 due to fear of weight gain (38, 39). Eating disorders in people with DM1 are associated with worse glycemic control, with elevated HbA1c (40–42).

There was an association between having more than 10 years of diagnosis and having increased HbA1c, while having less than 10 years of diagnosis was associated with having adequate HbA1c. These results are in line with those in the literature (32, 43–45). In response to these findings, it is understood that diabetes control may be more difficult over the years, due to increasing risks and treatment overload, such as the progressive reduction in the reserve of β cells and C-peptide secretion, and reduction of insulin sensitivity (46).

When analyzing the factors associated with adherence to Carbohydrate Counting and the HbA1c value, it was found that having adequate HbA1c was associated with having CC. Studies such as that by Donzeau et al. (20) and Kostopoulou et al. (21) show similar results, in which there were reductions in HbA1c levels when using the CC strategy. In the systematic review by Schmidt et al. (47) and in the systematic meta-analysis review by Vaz et al. (19), favorable results were also found for CC and HbA1c reduction. Thus, CC can be considered an important tool for glycemic control.

Regarding the association found between adequate HbA1c and practicing CC at lunch or dinner, it is hypothesized that most people should choose meals with a higher amount of carbohydrates to practice CC, for fear of having hypoglycemia or postprandial hyperglycemia (47, 48). Uliana et al. (23) found in their study, with adults with T1DM, that having more than six meals a day was associated with not performing CC. Furthermore, Fortin et al. (29) observed that finding it difficult to estimate the amount of carbohydrates before starting a meal was one of the greatest difficulties encountered by participants with T1DM with the practice of CC. Therefore, it is known that performing CC demands a high response cost, as behaviors such as identifying and calculating all carbohydrate-containing foods and estimating portion sizes are necessary, which reinforces the hypothesis that the patients can choose to do CC only in meals with higher amounts of carbohydrates (7, 12). However, the monitoring of carbohydrate intake by people with T1DM at all meals is essential to maintain control of bolus insulin doses, which requires discipline, but, consequently, helps in the glycemic control and nutritional status of these patients (29, 47).

As for the use of apps and the food scale, using specific and non-specific CC apps and using the food scale to do the CC, when eating something different than usual and to make culinary recipes was associated with having adequate HbA1c in this study. Using mobile apps and food scales helps to perform CC more accurately and frequently, optimizing glycemic control and reducing counting errors (49–51). In the cross-sectional study by Trawley et al. (52), the use of apps among people with T1DM to perform CC was associated with a lower self-reported HbA1c value and a higher frequency of glucose monitoring, corroborating the result found in the present study.

Regarding the associations between the monitoring with health professionals and the value of HbA1c, it was observed that having learned to do CC with a nutritionist or another health professional was associated with having adequate HbA1c. The SBD and the ADA affirm the need for a nutritionist experienced in the treatment of diabetes, with the purpose of carrying out specific training with patients, teaching them to measure and/or estimate the size of food portions, in addition to establishing the amount of carbohydrates from meals, through an individualized food plan (4, 7). Furthermore, undergoing medical nutrition therapy with qualified nutritionists is associated with reductions of 1.0% and 1.9% in HbA1c in adults with T1DM (53). Therefore, the present result emphasizes the importance of regular monitoring with a nutritionist and reinforces the relevance that other health professionals can exercise in the treatment of diabetes.

Regarding the associations between understanding the concepts of CC and HbA1c values, it is noted that knowing and knowing how to define most of the concepts was associated with having adequate HbA1c, while not mastering the concepts was associated with increased HbA1c. To date, no studies have been found that directly assess these relationships. However, in Brazil, there are already manuals available on the internet (e.g. 11,12) that explain the definitions of each concept. Thus, it is hypothesized that easy access to these materials can contribute to a better understanding. As a result, it is estimated that a better understanding favors adherence to CC and, as a result, patients have better HbA1c values.

On the other hand, having adequate HbA1c was associated with considering that not knowing what the insulin/carbohydrate ratio is does not influence the lack of adherence to CC. No studies were found that evaluated these variables either, however, it is assumed that with the creation and improvement of new technologies, such as mobile applications for CC, patients are able to perform the necessary calculations more easily, not needing to apply the concept at the time of use. In the systematic review by Dantas et al. (50) it was concluded that the use of CC applications contributes to better glycemic control in patients with T1DM, which is confirmed with the reduction of HbA1c.

In the present study, practicing CC and having been diagnosed for less than 10 years were identified as predictors of having adequate HbA1c, and practicing CC was a predictive factor for having adequate HbA1c, regardless of the time of diagnosis. Participants who were practicing CC were 3,273 times more likely to have adequate HbA1c, which is in agreement with studies that show that adherence to CC reduces HbA1c values in people with T1DM (16, 20, 21, 43, 53, 54). As for having a shorter diagnostic time being related to having adequate HbA1c, there is already a vast literature that reinforces this result and shows that over the years, HbA1c tends to increase (32, 43–45).

### 4.1 Limitations

It is important to emphasize that this study presents as a limitation the carrying out of the research in digital format, with the form being sent directly to people with T1DM via social networks, which may have caused a bias in the study population, with the exclusion of people without access to social networks. Based on the previously mentioned limitation, it is also necessary to highlight the difficulty of directly verifying whether the participants actually ate certain meals. Data collection on food consumption was based on participants’ self-reports through the online form, which can present challenges in terms of accuracy and reliability of the information provided.

Another limitation is the use of BMI as a parameter to assess malnutrition, considering that the comprehensive definition of malnutrition must consider factors other than BMI, such as medical history, complementary exams, and individualized clinical evaluation. However, an online survey has restrictions on the collection of detailed clinical data, such as laboratory tests and face-to-face clinical evaluation. Therefore, the use of BMI as an initial criterion for assessing malnutrition was a specific methodological decision, considering the inherent limitations of a survey carried out in digital format.

However, no studies were found that evaluated these particularities of the practice of CC associated with HbA1c in adults with T1DM in Brazil, which makes this study unprecedented and important insofar as it can encourage the conduct of new research. Therefore, it is necessary to carry out other studies that investigate these factors in larger samples and with a more homogeneous distribution of participants, in addition to more detailed approaches and more accurate data collection methods to investigate dietary patterns and their relationship with control glucose in this population.

## 5 Conclusion

It was concluded from the study that cross-sectional variables to CC, such as carrying it out in large meals, using applications and food scales, knowing and knowing how to define the concepts and learning the strategy with a nutritionist or other health professional, favor HbA1c values adequate in adults with T1DM. In addition, practicing CC and having a diagnosis time of less than 10 years were predictive factors of having adequate HbA1c.

Therefore, implementing CC in the treatment of people with T1DM is of fundamental importance in order to achieve glycemic control. For this, the need for the monitoring presence of the entire health team is reiterated, in order to achieve better adherence.

## Data availability statement

The original contributions presented in the study are included in the article/supplementary material. Further inquiries can be directed to the corresponding author.

## Ethics statement

The studies involving human participants were reviewed and approved by Research Ethics Committee from the Nucleus of Tropical Medicine of the Federal University of Pará - opinion n. 5.077.488. The patients/participants provided consent to participate in this study.

## Author contributions

Conceptualization, GU, LC, CP, JC and DG. Methodology, GU, CP and DG. Formal analysis, DG. Investigation, GU, LC, CP and DG. Writing—original draft preparation, GU, LC, CP, JC and DG. Writing—review and editing, GU, LC, CP, JC and DG. Visualization, GU, LC, CP, JC and DLG. Supervision, CP and DG. All authors contributed to the article and approved the submitted version.

## References

[1] International Diabetes Federation (IDF). IDF diabetes atlas (2021). Brussels, Belgium. Available at: https://www.diabetesatlas.org (Accessed December 05, 2022).

[2] American Diabetes Association Professional Practice Committee. 2. Classification and diagnosis of diabetes: Standards of Medical Care in Diabetes—2022. Diabetes Care (2022) 45(Suppl. 1):17–38. doi: 10.2337/dc18-S002 34964875

[3] Rodacki M Teles M Gabbay M Montenegro R Bertoluci M. Classificação do diabetes. Diretriz Oficial da Sociedade Bras Diabetes (2022) 1–28. doi: 10.29327/557753.2022-1

[4] American Diabetes Association Professional Practice Committee. 5. Facilitating behavior change and well-being to improve health outcomes: standards of medical care in diabetes—2022. Diabetes Care (2022) 45(Supplement_1):60–82. doi: 10.2337/dc22-S005 34964866

[5] Holt RIG DeVries JH Hess-Fischl A Hirsch IB Kirkman MS Klupa T. The management of type 1 diabetes in adults. A consensus report by the American Diabetes Association (ADA) and the European Association for the Study of Diabetes (EASD). Diabetes Care (2021) 44(11):2589–625. doi: 10.2337/dci21-0043 34593612

[6] Skyler JS Bakris GL Bonifacio E Darsow T Eckel RH Groop L. Differentiation of diabetes by pathophysiology, natural history, and prognosis. Diabetes (2017) 66(2):241–55. doi: 10.2337/db16-0806 PMC5384660 27980006

[7] Sociedade Brasileira de Diabetes (SBD) (Brazilian Society of Diabetes). Diretrizes Sociedade Brasileira de Diabetes 2019-2020 (Brazilian Diabetes Society Guidelines 2019–2020) (2019). São Paulo, Brasil: Clanad Editora Científica. Available at: https://www.diabetes.org.br/profissionais/images/DIRETRIZES-COMPLETA-2019-2020.pdf (Accessed December 05, 2022).

[8] American Diabetes Association [ADA]. 4. Lifestyle management. Diabetes Care (2017) 40(Supplement_1):33–43. doi: 10.2337/dc17-S007 27979891

[9] Argüello R Cáceres M Bueno E Benítez A Grijalba RF. Utilización del conteo de carbohidratos en la Diabetes Mellitus, in: ANALES de la Facultad de Ciencias Médicas (2013). Available at: http://scielo.iics.una.py/pdf/anales/v46n1/v46n1a05.pdf (Accessed December 08, 2022).

[10] Diabetes Control and Complications Trial Research Group. The effect of intensive treatment of diabetes on the development and progression of long-term complications in insulin-dependent diabetes mellitus. New Engl J Med (1993) 329(14):977–86. doi: 10.1056/NEJM199309303291401 8366922

[11] Sociedade Brasileira de Diabetes (SBD) (Brazilian Society of Diabetes). Manual de Contagem de Carboidratos para pessoas com diabetes (Carbohydrate Counting Manual for People with Diabetes) (2016). São Paulo, Brasil: Departamento de Nutrição da Sociedade Brasileira de Diabetes (Department of Nutrition of the Brazilian Society of Diabetes. Available at: https://diabetes.org.br/wp-content/uploads/2021/05/manual-de-contagem-de-carbo.pdf (Accessed December 08, 2022).

[12] Centro de Diabetes de Belo Horizonte (CDBH) (Belo Horizonte Diabetes Center). Manual de Contagem de Carboidratos (Carbohydrate Counting Manual). 5th ed. Belo Horizonte, Brasil: Novo Nordisk (2020) p. 1–160.

[13] Sociedade Brasileira de Diabetes (SBD) (Brazilian Society of Diabetes). Manual de nutrição: profissional da saúde (Nutrition manual: health professional). São Paulo, Brasil: Departamento de Nutrição e Metabologia (Department of Nutrition and Metabology (2009) p. 1–60.

[14] Sociedade Brasileira de Diabetes (SBD) (Brazilian Society of Diabetes). Manual Oficial de Contagem de Carboidratos para as Pessoas com Diabetes (Official Handbook of Carbohydrate Counting for People with Diabetes). Rio de Janeiro, Brasil: Departamento de Nutrição da Sociedade Brasileira de Diabetes (Department of Nutrition of the Brazilian Society of Diabetes (2009) p. 1–66.

[15] Franz MJ Powers MA Leontos C Holzmeister LA Kulkarni K Monk A. The evidence for medical nutrition therapy for type 1 and type 2 diabetes in adults. J Am Diet Assoc (2010) 110(12):1852–89. doi: 10.1016/j.jada.2010.09.014 21111095

[16] Tascini G Berioli MG Cerquiglini L Santi E Mancini G Rogari F. Carbohydrate counting in children and adolescents with type 1 diabetes. Nutrients (2018) 10(1):109. doi: 10.3390/nu10010109 29361766 PMC5793337

[17] Pititto B Dias M Moura F Lamounier R Calliari S Bertoluci M. Metas no tratamento do diabetes. Diretriz Oficial da Sociedade Bras Diabetes (2022), 1–23. doi: 10.29327/557753.2022-3

[18] American Diabetes Association Professional Practice Committee. 6. Glycemic targets: standards of medical care in diabetes—2022. Diabetes Care (2022) 45(Supplement_1):83–96. doi: 10.2337/dc22-S006 34964868

[19] Vaz EC Porfírio GJM Nunes HRC Nunes-Nogueira V. Effectiveness and safety of carbohydrate counting in the management of adult patients with type 1 diabetes mellitus: a systematic review and meta-analysis. Arch Endocrinol Metab (2018) 62(3):337–45. doi: 10.20945/2359-3997000000045 PMC10118793 29791661

[20] Donzeau A Bonnemaison E Vautier V Menut V Houdon L Bendelac N. Effects of advanced carbohydrate counting on glucose control and quality of life in children with type 1 diabetes. Pediatr Diabetes (2020) 21(7):1240–8. doi: 10.1111/pedi.13076 32644264

[21] Kostopoulou E Livada I Partsalaki I Lamari F Skiadopoulos S Gil APR. The role of carbohydrate counting in glycemic control and oxidative stress in patients with type 1 diabetes mellitus (T1DM). Hormones (Athens Greece) (2020) 19(3):433–8. doi: 10.1007/s42000-020-00189-8 32221838

[22] Ministério da Saúde (Ministry of Health). Resolução No 510, de 07 de abril de 2016 (Resolution No 510, of April 7, 2016), in: *Dispõe sobre as Normas Aplicáveis A Pesquisas em Ciências Humanas e Sociais Cujos Procedimentos Metodológicos Envolvam A Utilização de Dados Diretamente Obtidos Com os Participantes ou de Informações Identificáveis ou que Possam Acarretar Riscos Maiores do que os Existentes na vida Cotidiana (Provides for the Rules Applicable to Research in Human and Social Sciences Whose Methodological Procedures Involve the Use of Data Directly Obtained from the Participants or Identifiable Information or That May Entail Greater Risks than Those Existing in the Everyday Life)* (2016). Diário Of. União. Available at: https://pesquisa.in.gov.br/imprensa/jsp/visualiza/index.jsp?jornal=1&data=24/05/2016&pagina=44 (Accessed June 21, 2021).

[23] Uliana GC Carvalhal MML Berino TN Reis AL Felício KM Felício JS. Adherence to carbohydrate counting improved diet quality of adults with type 1 diabetes mellitus during social distancing due to COVID-19. Int J Environ Res Public Health (2022) 19(16):9776. doi: 10.3390/ijerph19169776 36011412 PMC9408385

[24] Lobiondo-Wood G Haber J. Desenhos não-experimentais. In: Lobiondo-Wood G Haber J, editors. Pesquisa em enfermagem: métodos, avaliação crítica e utilização, 4 ed. Rio de Janeiro, Brazil: Guanabara-Koogan (2001). p. 110–21.

[25] Polit DF Beck CT. Fundamentos de pesquisa em enfermagem: avaliação de evidências para a prática da enfermagem. 7th ed. Porto Alegre, Brazil: Artmed Editora (2011). p. 670.

[26] Ministério da Saúde (Ministry of Health). Resolução No 466, de 12 de dezembro de 2012 (Resolution No 466, of December 12, 2012), in: Dispõe sobre as diretrizes e normas para pesquisas com seres humanos (Provides for guidelines and standards for research with human beings) (2012). Diário Oficial da União. Available at: http://conselho.saude.gov.br/resolucoes/2012/Reso466.pdf (Accessed July 04, 2021).

[27] Ministério da Saúde (Ministry of Health). *Vigitel Brasil 2021: vigilância de fatores de risco e proteção para doenças crônicas por inquérito telefônico: estimativas sobre frequência e distribuição sociodemográfica de fatores de risco e proteção para doenças crônicas nas capitais dos 26 estados brasileiros e no Distrito Federal em 2021 (Vigitel Brazil 2021: surveillance of risk and protective factors for chronic diseases by telephone survey: estimates of frequency and sociodemographic distribution of risk and protective factors for chronic diseases in the capitals of the 26 Brazilian states and the Federal District in 2021)* (2021). Brasília: Ministério da Saúde. Available at: https://www.gov.br/saude/pt-br/centrais-de-conteudo/publicacoes/publicacoes-svs/vigitel/vigitel-brasil-2021-estimativas-sobre-frequencia-e-distribuicao-sociodemografica-de-fatores-de-risco-e-protecao-para-doencas-cronicas/ (Accessed January 12, 2023).

[28] Maahs DM West NA Lawrence JM Mayer-Davis EJ. Epidemiology of type 1 diabetes. Endocrinol Metab Clinics North America (2010) 39(3):481–97. doi: 10.1016/j.ecl.2010.05.011 PMC2925303 20723815

[29] Fortin A Rabasa-Lhoret R Roy-Fleming A Desjardins K Brazeau AS Ladouceur M. Practices, perceptions and expectations for carbohydrate counting in patients with type 1 diabetes – Results from an online survey. Diabetes Res Clin Pract (2017) 126:214–21. doi: 10.1016/j.diabres.2017.02.022 28273644

[30] Kemp S. Digital 2022: Brazil, in: Kepios (2022). Available at: https://datareportal.com/reports/digital-2022-Brazil?rq=Brazil (Accessed January 12, 2023).

[31] Bell KJ Barclay AW Petocz P Colagiuri S Brand-Miller JC. Efficacy of carbohydrate counting in type 1 diabetes: a systematic review and meta-analysis. Lancet Diabetes Endocrinol (2014) 2(2):133–40. doi: 10.1016/S2213-8587(13)70144-X 24622717

[32] Joseph M Shyamasunder AH Gupta RD Anand V Thomas N. Demographic details, clinical features, and nutritional characteristics of young adults with Type 1 diabetes mellitus - A South Indian tertiary center experience. Indian J Endocrinol Metab (2016) 20(6):799–804. doi: 10.4103/2230-8210.192895 27867883 PMC5105564

[33] Tani N Michiue T Chen JH Oritani S Ishikawa T. Usefulness of postmortem biochemistry in identification of ketosis: Diagnosis of ketoacidosis at the onset of autoimmune type 1 diabetes in an autopsy case with cold exposure and malnutrition. Legal Med (2016) 22:23–9. doi: 10.1016/j.legalmed.2016.07.006 27591535

[34] Yanase T Yanagita I Muta K Nawata H. Frailty in elderly diabetes patients. Endocrine J (2018) 65(1):1–11. doi: 10.1507/endocrj.EJ17-0390 29238004

[35] Davidson J. Diabulimia: how eating disorders can affect adolescents with diabetes. Nurs standard (Royal Coll Nurs (Great Britain): 1987) (2014) 29(2):44–9. doi: 10.7748/ns.29.2.44.e7877 25204951

[36] Winston AP. Eating disorders and diabetes. Curr Diabetes Rep (2020) 20(8):1–6. doi: 10.1007/s11892-020-01320-0 32537669

[37] Coleman SE Caswell N. Diabetes and eating disorders: an exploration of ‘Diabulimia’. BMC Psychol (2020) 8(1):101. doi: 10.1186/s40359-020-00468-4 32967730 PMC7513317

[38] Goebel-Fabbri AE Anderson BJ Fikkan J Franko DL Pearson K Weinger K. Improvement and emergence of insulin restriction in women with type 1 diabetes. Diabetes Care (2011) 34(3):545–50. doi: 10.2337/dc10-1547 PMC3041178 21266653

[39] Balfe M Doyle F Smith D Sreenan S Conroy R Brugha R. Dealing with the devil: weight loss concerns in young adult women with type 1 diabetes. J Clin Nurs (2013) 22(13-14):2030–8. doi: 10.1111/jocn.12231 23745648

[40] Young V Eiser C Johnson B Brierley S Epton T Elliott J. Eating problems in adolescents with Type 1 diabetes: a systematic review with meta-analysis. Diabetic Med (2013) 30(2):189–98. doi: 10.1111/j.1464-5491.2012.03771.x 22913589

[41] Scheuing N Bartus B Berger G Haberland H Icks A Knauth B. Clinical characteristics and outcome of 467 patients with a clinically recognized eating disorder identified among 52,215 patients with type 1 diabetes: a multicenter german/Austrian study. Diabetes Care (2014) 37(6):1581–9. doi: 10.2337/dc13-2156 24623022

[42] Goebel-Fabbri AE Fikkan J Franko DL Pearson K Anderson BJ Weinger K. Insulin restriction and associated morbidity and mortality in women with type 1 diabetes. Diabetes Care (2008) 31(3):415–9. doi: 10.2337/dc07-2026 18070998

[43] Fortins RF Lacerda EMA Silverio RNC do Carmo CN Ferreira AA Felizardo C. Predictor factors of glycemic control in children and adolescents with type 1 diabetes mellitus treated at a referral service in Rio de Janeiro, Brazil. Diabetes Res Clin Pract (2019) 154:138–45. doi: 10.1016/j.diabres.2019.05.027 31150723

[44] Galler A Lindau M Ernert A Thalemann R Raile K. Associations between media consumption habits, physical activity, socioeconomic status, and glycemic control in children, adolescents, and young adults with type 1 diabetes. Diabetes Care (2011) 34(11):2356–9. doi: 10.2337/dc11-0838 PMC3198300 21926289

[45] Osan JK Punch JD Watson M Chan YX Barrie P Fegan PG. Associations of demographic and behavioural factors with glycaemic control in young adults with type 1 diabetes mellitus. Internal Med J (2016) 46(3):332–8. doi: 10.1111/imj.12991 26748888

[46] Zaharia OP Strassburger K Strom A Bönhof GJ Karusheva Y Antoniou S. Risk of diabetes-associated diseases in subgroups of patients with recent-onset diabetes: a 5-year follow-up study. Lancet Diabetes Endocrinol (2019) 7(9):684–94. doi: 10.1016/S2213-8587(19)30187-1 31345776

[47] Schmidt S Schelde B Nørgaard K. Effects of advanced carbohydrate counting in patients with type 1 diabetes: a systematic review. Diabetic medicine: J Br Diabetic Assoc (2014) 31(8):886–96. doi: 10.1111/dme.12446 24654856

[48] Brazeau AS Mircescu H Desjardins K Leroux C Strychar I Ekoé JM. Carbohydrate counting accuracy and blood glucose variability in adults with type 1 diabetes. Diabetes Res Clin Pract (2013) 99(1):19–23. doi: 10.1016/j.diabres.2012.10.024 23146371

[49] Alfonsi JE Choi EEY Arshad T Sammott SS Pais V Nguyen C. Carbohydrate counting app using image recognition for youth with type 1 diabetes: pilot randomized control trial. JMIR MHealth UHealth (2020) 8(10):e22074. doi: 10.2196/22074 33112249 PMC7657721

[50] Dantas NS Albuquerque NV Rebouças Moreira T Bezerrra AN Ramos LTT Rebouças KSC. Use of application for carbohydrates counting as a tool to help in the self-management of type 1 diabetes mellitus: a systematic review. Research Soc Dev (2023) 12(1):1–10. doi: 10.33448/rsd-v12i1.39270

[51] Oliveira FMD Calliari LEP Feder CKR Almeida MFOD Pereira MV Alves MTTAF. Efficacy of a glucose meter connected to a mobile app on glycemic control and adherence to self-care tasks in patients with T1DM and LADA: a parallel-group, open-label, clinical treatment trial. Arch Endocrinol Metab (2021) 65(2):185–97. doi: 10.20945/2359-3997000000334 PMC10065322 33905630

[52] Trawley S Baptista S Browne JL Pouwer F Speight J. The use of mobile applications among adults with type 1 and type 2 diabetes: results from the second MILES-Australia (MILES-2) study. Diabetes Technol Ther (2017) 19(12):730–8. doi: 10.1089/dia.2017.0235 29028442

[53] Franz MJ MacLeod J Evert A Brown C Gradwell E Handu D. Academy of nutrition and dietetics nutrition practice guideline for type 1 and type 2 diabetes in adults: systematic review of evidence for medical nutrition therapy effectiveness and recommendations for integration into the nutrition care process. J Acad Nutr Dietetics (2017) 117(10):1659–79. doi: 10.1016/j.jand.2017.03.022 28533169

[54] Fu S Li L Deng S Zan L Liu Z. Effectiveness of advanced carbohydrate counting in type 1 diabetes mellitus: a systematic review and meta-analysis. Sci Rep (2016) 6:37067. doi: 10.1038/srep37067 27841330 PMC5107938

